# Comparing mapping and direct hyperspectral imaging in stand‐off Raman spectroscopy for remote material identification

**DOI:** 10.1002/jrs.5607

**Published:** 2019-04-30

**Authors:** Christoph Gasser, María González‐Cabrera, María José Ayora‐Cañada, Ana Domínguez‐Vidal, Bernhard Lendl

**Affiliations:** ^1^ Institute of Chemical Technologies and Analytics TU Wien Vienna Austria; ^2^ Department of Physical and Analytical Chemistry Universidad de Jaén Jaén Spain

**Keywords:** stand‐off, remote detection, Raman spectroscopy, hyperspectral imaging

## Abstract

Stand‐off Raman spectroscopy offers a highly selective technique to probe unknown substances from a safe distance. Often, it is necessary to scan large areas of interest. This can be done by pointwise imaging (PI), that is, spectra are sequentially acquired from an array of points over the region of interest (point‐by‐point mapping). Alternatively, in this paper a direct hyperspectral Raman imager is presented, where a defocused laser beam illuminates a wide area of the sample and the Raman scattered light is collected from the whole field of view (FOV) at once as a spectral snapshot filtered by a liquid crystal tunable filter to select a specific Raman shift. Both techniques are compared in terms of achievable FOV, spectral resolution, signal‐to‐noise performance, and time consumption during a measurement at stand‐off distance of 15 m. The HSRI showed superior spectral resolution and signal‐to‐noise ratio, while more than doubling the FOV of the PI at laser power densities reduced by a factor of 277 at the target. Further, the output hyperspectral image data cube can be processed with state of the art chemometric algorithms like vertex component analysis in order to get a simple deterministic false color image showing the chemical composition of the target. This is shown for an artificial polymer sample, measured at a distance of 15 m.

## 1 INTRODUCTION

Stand‐off Raman spectroscopy is a highly versatile remote detection technique, which combines the advantages of Raman spectroscopy with the benefit of separating physically the instrumentation from the sampling point. Illumination via laser light and collection of primarily backscattered Raman photons allow for a remote detection scheme, as long as free propagation of photons is possible. This makes stand‐off Raman spectroscopy a potent analytical solution for a variety of applications. The exploration of different materials located on planetary surfaces is one of the most prominent studied application possibilities, given its advantages in terms of accessibility of remote objects also when the rover is stationary.^1^ A wide range of samples, such as minerals, organics, and inorganics have been detected before by using remote Raman instruments.^2^, ^3^, ^4^ The possibility of maintaining a distance from the target while obtaining relevant analytical information without compromising the safety of the operator or the instrument makes stand‐off Raman spectroscopy also the ideal tool for detection of hazardous or harmful materials. This has been demonstrated with different types of explosives^5^, ^6^ and possible concealed threats in several kind of containers.^7^, ^8^ Moreover, art, heritage, and restoration applications could also benefit from the use of stand‐off instrumentation, due to the considerable reduction of the risk of producing contaminations or alterations in the composition of original works of artistic value and the improved accessibility to hard‐to‐reach areas such as ceilings or high walls.

Hyperspectral imaging describes a wide range of increasingly used and constantly evolving techniques for chemical and structural analysis, which provides both spatial and spectral information for a given sample of interest.^9^, ^10^ The result of these measurements is a three‐dimensional dataset, which consists of the two‐dimensional spatial information and an additional spectral dimension, thus forming a hyperspectral image (HSI) cube. Generally, two different techniques of obtaining this HSI cube can be differentiated: spatial and spectral methods. Regarding spatial scanning methods, two different approaches can be distinguished: point‐by‐point mapping (whiskbroom imaging) and line scanning (pushbroom imaging).^11^ In whiskbroom imaging, a spectrum of a specific spatial position of the sample is obtained, and then the area of interest is scanned by moving pointwise to generate a mapping of the sample surface. Most of Raman‐based hyperspectral imaging techniques use microscopes and accurate translation stages to move the sample in order to achieve Raman maps.^12^ If however, a whole line of spectra is recorded at the same time and is scanned laterally over the sample a HSI can be created much faster. This is generally known as pushbroom approach, which utilizes the fact that most detectors used for Raman spectroscopy are two‐dimensional arrays with one free dimension, which in turn can be used to image one dimension of the sample surface onto the detector thus considerably increasing the acquisition speed.^9^ On the other hand, spectral scanning methods collect a two‐dimensional spatial image for each wavelength band at a time. Therefore, the HSI cube is built by stacking spectral snapshots on top of each other. This requires the possibility of selecting a certain wavelength and image it onto the detector array, which can be facilitated by tunable bandpass filters. Such filters have multiple prerequisites, they have to provide an adequate width of the bandpass in order to ensure sufficient spectral resolution, good transmission characteristics, and large optical aperture for passing the image and fast tuning. Among several possible technologies, acusto‐optical tunable filters (AOTFs), tunable Fabry–Perot cavities, and liquid crystal tunable filters (LCTFs) are the most commonly used.^13^ AOTFs, which usually consist of a birefringent crystal attached to a piezoelectric transducer, provide a programmable grating, thus acting like a tunable filter. The transmitted light is diffracted by the acoustic wave, and the spectral position of the bandpass is selected by changing the frequency of the transducer, that is, the sound wave.^14^ The main drawbacks of AOTFs are the image degradation due to image shift with tuning, image dispersion in the diffraction direction, and the bigger bandwidth compared with LCTFs.^15^ Tunable Fabry–Perot cavities consist of two reflective surfaces, which is transmission characteristics depend on, among others, the distance between the surfaces.^16^ Piezoelectric crystals can be used to facilitate the spectral tunability of TPFCs with large optical apertures by changing the distance of the reflective surfaces.^17^ LCTFs are based on Lyot filters, where a filter stage is based upon one birefringent crystal plate sandwiched by orthogonal polarizers.^18^ Multiple stacks with varying thickness of plate retarders create a narrowband bandpass, which can be tuned by using a liquid crystal panel.^19^, ^20^ Since LCTFs offer fast and reliable tuning with good spectral bandwidth at a small footprint, they have been used for several HSI applications,^21^ among them Raman spectroscopic imagers.^22^

In this study, we compare the performance of two different stand‐off Raman imaging approaches: a pointwise or whiskbroom imager (PI), where the HSI is created by scanning the laser point over the sample, and a direct hyperspectral Raman imaging (HSRI), where the HSI is created by stacking spectral snapshots on top of each other. The PI system employs a classical diffracting spectrometer, coupled to the telescope by an optical fiber with the laser point being scanned over the sample by means of an electronically controlled mirror. The HSRI uses a LCTF to select a specific Raman shift and collect a spectral image, while the laser illuminates the whole field of view (FOV) of the telescope or rather the camera.^23^ In order to suppress unspecific light (originating from, e.g., daylight), the emission of the laser pulse and the acquisition of the backscattered photons were synchronized. Both configurations are compared in terms of achievable FOV, signal‐to‐noise ratio (SNR) and time consumption during a measurement at a stand‐off distance of 15 m. Furthermore, the importance of spatial resolution is explored for the purpose of chemical identification of small amounts of substances at stand‐off distances.

## 2 MATERIALS AND METHODS

**Figure 1:**
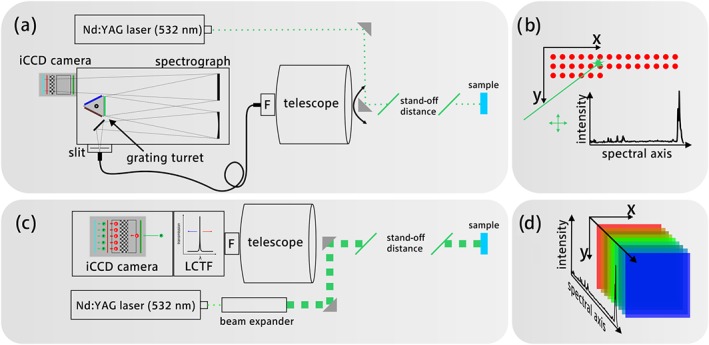
Simplified sketch of the instrumentation used in this study for (a) pointwise stand‐off Raman spectroscopy and (c) direct stand‐off Raman imaging setup with an illustration of the hyperspectral image generation for both methods respectively in (b) and (d) [Colour figure can be viewed at wileyonlinelibrary.com]

In this study, two different optical setups were employed, which are displayed in Figure 1a,c. The first includes a collimated laser beam creating an illuminated point with a diameter of 6 mm, which is mapped over the target area with the help of a motorized mirror. The whole Raman spectrum is collected from each measurement point and then combined into a HSI cube (Figure 1b). The second setup employs a widened and unfocused laser beam, which illuminates an area with an apparent diameter of approximately 100 mm. The scattered Rayleigh light is filtered and directly imaged onto the camera, thus generating the HSI cube, one image at a time, along the spectral axis (Figure 1d).

In both systems, a Q‐switched, frequency doubled (532 nm) Nd:YAG NL301HT laser (EKSPLA, Lithuania) with a pulse energy of 50 mJ, a pulse length of 4.4 ns, and a repetition rate of 10 Hz was used as an excitation source. The beam profile is specified exhibiting a top‐hat beam profile in the near field and a near‐Gaussian profile in the far field with a beam divergence lower than 0.6 mrad.

### 2.1 Pointwise stand‐off Raman imager (PI)

For the pointwise stand‐off Raman system, the laser was aligned coaxially to a 6″ Schmidt‐Cassegrain telescope (C6‐A‐XLT, f = 1.5 m, f/9.9, Celestron, USA) for the collection of Raman scattered light using a motorized kinematic mirror mount (KS1‐Z8, Thorlabs, USA), which allowed for the mapping of the laser onto the sample area. The backscattered Rayleigh light was filtered using a long pass filter (LP03‐532RE, cut‐off wavelength 533.3 nm, OD > 6, Semrock, USA), and the Raman photons were guided to an Acton standard series SP‐2750 spectrograph (slit 120 μm, f/10, 300 grooves/mm, Princeton Instruments, Germany) via a round‐to‐slit fiber optical bundle cable consisting of nineteen 200‐μm diameter optical fibers (FCRL 19UV200, NA 0.22, Avantes, Netherlands). To match the F number of telescope and fiber bundle, a f = 50‐mm lens was used. Finally, the backscattered light was detected by a PIMAX 1024RB intensified charge‐coupled device (iCCD) camera (QE 7.5% @ 600 nm, Princeton Instruments, USA). The outgoing laser pulse and the gate of the iCCD camera were synchronized so that the measurement window coincided with the maximum Raman signal. Data acquisition and mapping was automated using LabVIEW® (National Instruments, USA).

### 2.2 Direct stand‐off hyperspectral Raman imager (HSRI)

The direct stand‐off imager (Figure 1c) employed an expanded laser beam, generated using a defocused Galilean‐type beam expander in order to achieve an apparent beam diameter of approximately 100 mm at the sample. The backscattered Rayleigh photons were again filtered through a long pass filter (LP03‐532RE, cut‐off wavelength 533.3 nm, OD > 6, Semrock, USA). Subsequently, the Raman photons were filtered using a tunable LCTF filter (VariSpec VISR, Perkin‐Elmer, USA) with a spectral resolution of 0.25 nm and directly imaged onto an iCCD equipped with a quadratic sensor (PIMAX 4 1024f‐HBf iCCD. 1,024 × 1,024 pixels, 13‐μm pixels, QE 45% @ 600 nm, Princeton Instruments, USA). The VariSpec LCTF is of the Evans Split element variety, which requires only half as many polarizers as an equivalent Lyot filter type.^24^ The LCTF has a clear aperture of 20 mm, a free spectral range of 480–720 nm, an angle of acceptance of 7° and a response time of 150 ms. The transmission of the LCTF varies over the whole spectral range, with a mean transmission of 22.5% in the area of interest. Each acquired spectral image was stacked in order to build the hyperspectral data cube. This process was automated using LabVIEW® (National Instruments, USA).

### 2.3 Chemometric methods

Vertex component analysis (VCA) was used for the evaluation of the generated HSI data cubes. VCA is a technique for unsupervised endmember extraction assuming the data is a linear mixture of pure components, also called endmembers.^25^ For the analysis, the commercial software ImageLab (Epina GmbH, Austria) was used.

### 2.4 Chemicals

Plates of different chemical composition and thickness were acquired from RS Components (United Kingdom), namely, polypropylene (PP, 2 mm), polyethylene (PE, 4 mm), polytetrafluoroethylene (PTFE or Teflon, 6 mm), and nylon (N6, 4 mm). Pieces were cut out and glued together using small amounts of cyanoacrylate on the edges of the cut pieces. Sulfur (>99.98%) was obtained from Sigma‐Aldrich and for the spatial resolution experiments, polylactic acid plates were designed as samples containers on the computer and 3D printed afterwards.

## 3 RESULTS AND DISCUSSION

### 3.1 Illumination system

**Figure 2:**
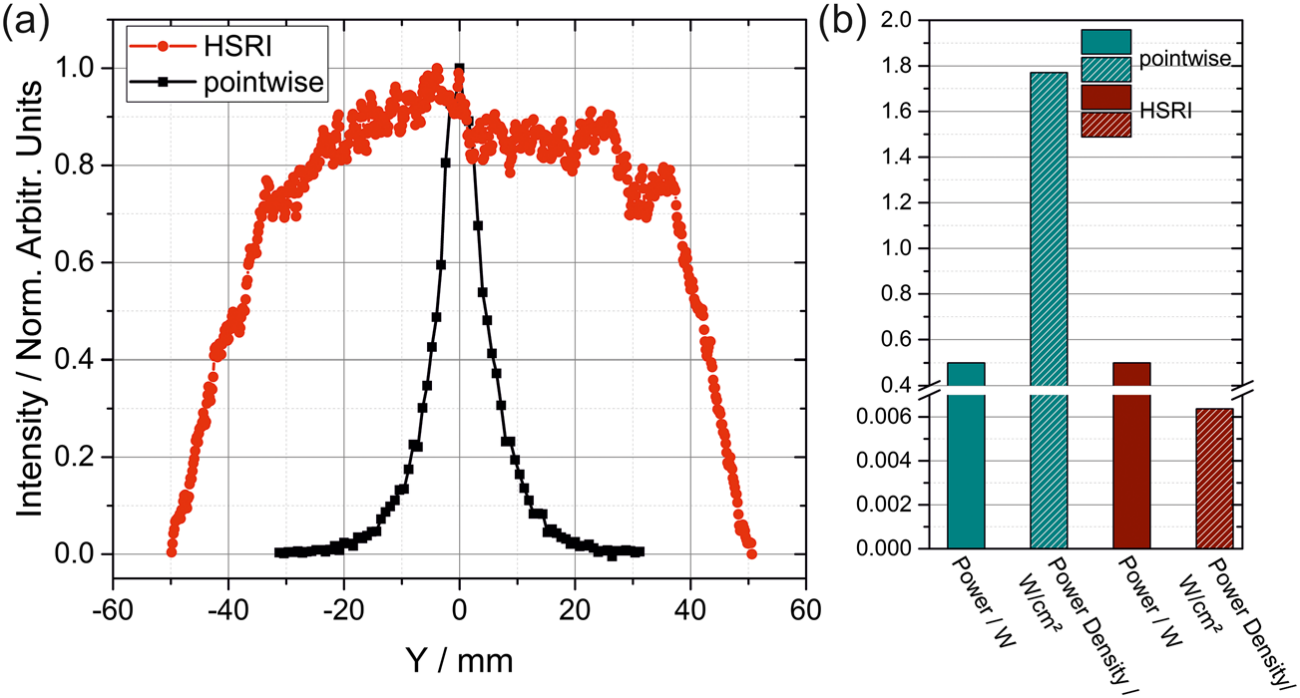
(a) Measured field of view for both configurations on the horizontal axis Y. (b) Laser power and power density used in this study for both configurations [Colour figure can be viewed at wileyonlinelibrary.com]

Illumination and FOV differ greatly between the two systems. Figure 2a depicts the FOV of both configurations over the horizontal axis Y, measured on a PP plate of 2 mm thickness at a distance of 15 m. For the PI setup, this was done by scanning the laser point laterally step by step away from the center and recording a spectrum at every point. Afterwards, the intensity of the band at 2,890 cm^−1^ was assessed in dependence of the horizontal position of the illumination point. The resulting curve (black) shows a distinct fall off with increasing offset from the central point, reaching the 10% mark at a diameter of approximately 24 mm. To assess the FOV of the HSRI prototype, the same PP plate was used at 15 m distance as a target. A spectral image at 2,890 cm^−1^ was acquired, and the intensity was evaluated along the same horizontal axis Y as before. The HSRI configuration shows a broader intensity profile, mainly influenced by the emission characteristics of the laser beam. Normally, the FOV of a telescope and CCD chip combination is influenced by the focal length of the telescope, the stand‐off distance, and the size of the CCD chip itself. For the employed HSRI system, this surmounts to a theoretical FOV of approximately 110 m. Ideally, for the HSRI the laser beam has an ideal top‐hat structure also in the far field, which means the energy density within the beam is uniform. This leads to a uniform intensity distribution in aberration‐free optical systems, which enables the usage of the whole image without loss of SNR in less illuminated border regions. For the HSRI presented in this study, the effective FOV with adequate Raman intensity amounts to a diameter of approximately 70 mm.

In the PI system, the numerical aperture and the diameter of the fiber bundle limit the FOV. In contrast to the HSRI, the PI system with its fiber bundle with a numerical aperture of 0.22 and a diameter of 1.5 mm, the effective FOV is reduced to an area with a diameter of 24 mm. This means that the maximal useful area that can be illuminated for the PI is 452 mm^2^ and for the HSRI it is 3,848 mm^2^, a less than 10‐fold increase.

### 3.2 Spectral investigations

**Figure 3:**
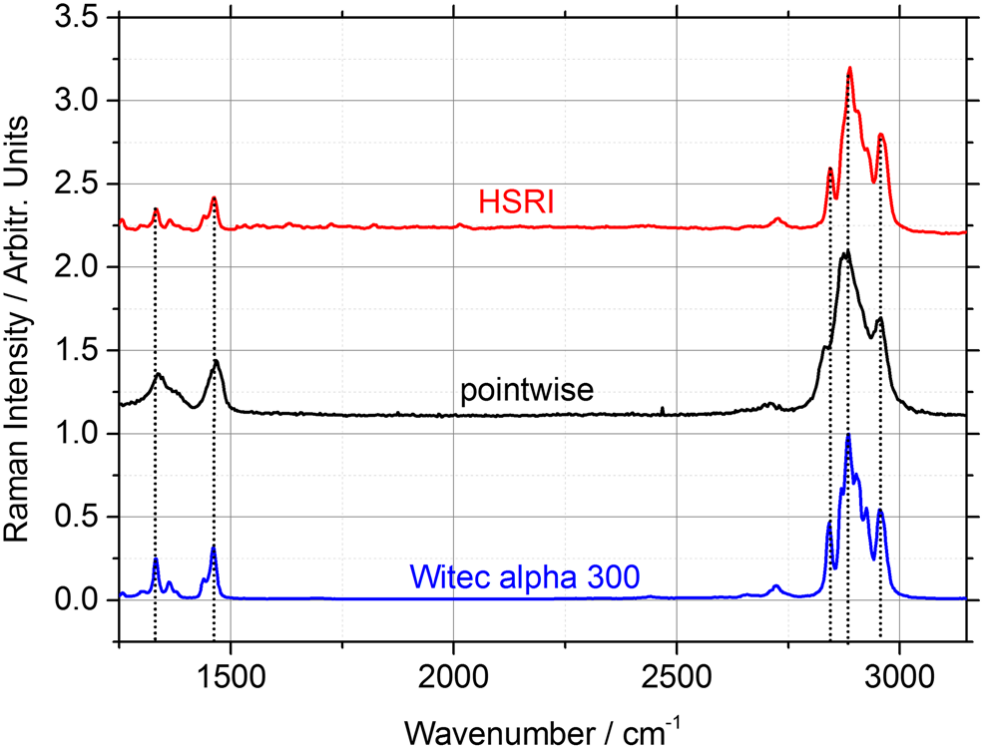
Exemplary spectra of polypropylene (PP) obtained with the hyperspectral Raman imaging (HSRI) system (red) and with the pointwise stand‐off imager (black) at 15‐m distance. Reference PP spectrum (blue) taken with a commercial instrument (Witec alpha300rsa) [Colour figure can be viewed at wileyonlinelibrary.com]

Spectra obtained with both systems were compared with spectra taken with a commercially available Raman microscope (alpha300rsa, Witec, Germany) with the same excitation wavelength of 532 nm used throughout this study. Again, a PP plate was the target of choice, and Figure 3 shows the obtained spectra.

On the high Raman shift range from 2,840 to 2,950 cm^−1^ typical bands arising from the symmetric and asymmetric stretch vibrations of the methyl and methylene group can be found. Additionally, the bands attributed to the symmetric and asymmetric bending of the methyl and methylene group present in PP are visible between 1,260 and 1,500 cm^−1^ in the chosen spectral range. Both configurations of stand‐off Raman imagers show the same bands as the reference taken by the Raman microscope, and the spectral positions are in good agreement. The biggest difference in the shown spectra is their respective resolution. The Raman microscope uses a 600 gr/mm grating, which results in a spectral resolution of approximately 4 cm^−1^ with the spectrograph and camera built into the instrument. The PI system with a 300 gr/mm grating and a 750‐mm focal length f/10 spectrograph has the lowest spectral resolution of the compared systems with approximately 15 cm^−1^. The HSRI imager does not use a classical spectrograph as the dispersive element but the LCTF. Hence, the spectral resolution is a function of the width of the transmission curve, which is specified by the manufacturer to be constant over the complete visible range at 0.25 nm. This amounts to a mean spectral resolution of 7.4 cm^−1^ in the observed spectral range. Additionally, the relative intensities vary between spectrograph and filter systems. The tunable filter has lower throughput at shorter wavelengths, which becomes apparent when comparing bands at higher Raman shifts with bands at lower Raman shifts for the spectrograph and the filter configuration in Figure 3. Nevertheless, the resolution is sufficient for most common applications, even exceeding the PI setup, which already showed its usefulness in previous works.^4^

The tunable filter in the HSRI is mainly used because of the ability to perform direct imaging. Hence, in this system, it is important to evaluate the uniformity of the transmission window in terms of spectral position and spectral resolution over the FOV.

To do this, a PTFE plate with an appropriate size was measured using the HSRI prototype. The region from 700 to 1,500 cm^−1^ with spectral steps of 2 cm^−1^ was acquired, thereby oversampling the theoretical resolution of the HSRI of approximately 7 cm^−1^. PTFE shows several bands in this region, the two most important ones are the skeletal stretching at approximately 746 cm^−1^ and the symmetric CF_2_ stretching modes at approximately 1,380 cm^−1^.^26^ Since the band at 746 cm^−1^ shows higher intensity and narrow linewidth, it was chosen to be the indicator for spectral resolution and position accuracy of the filter. To assess these parameters, a fit of the band using a pseudo‐Voigt profile^27^ was performed, and the full width at half maximum (FWHM) and the center of the fitted function were determined. Figure S3b displays an exemplary fit. This was done for the whole HSI cube, the distribution of the center position and FWHM over the imaged surface are represented in Figure S3c,d, respectively. In the central circle with a diameter of 70 mm, the center position varies around 746 cm^−1^ by no more than 2 cm^−1^ with a standard deviation of 0.28 cm^−1^. An aggregation of extreme values at the edges of the image is observed. Similarly, the calculated FWHM ranges from 10 to 14 cm^−1^ in the inner circle, with considerable outliers at the edges of the image. These extreme values are due to the lower intensity of the exciting laser at the edges of the imaged area, reducing the SNR of the resulting spectra and preventing a regular fit for center position and especially for FWHM evaluation. Overall, the reported deviations of both center position and FWHM are within the expected error margin, given that the spectral resolution of the filter is specified to be around 7.5 cm^−1^. This means that adequate Raman spectra collection using the tunable filter is viable for the whole aperture given a strong enough laser excitation

### 3.3 Signal to noise

**Figure 4:**
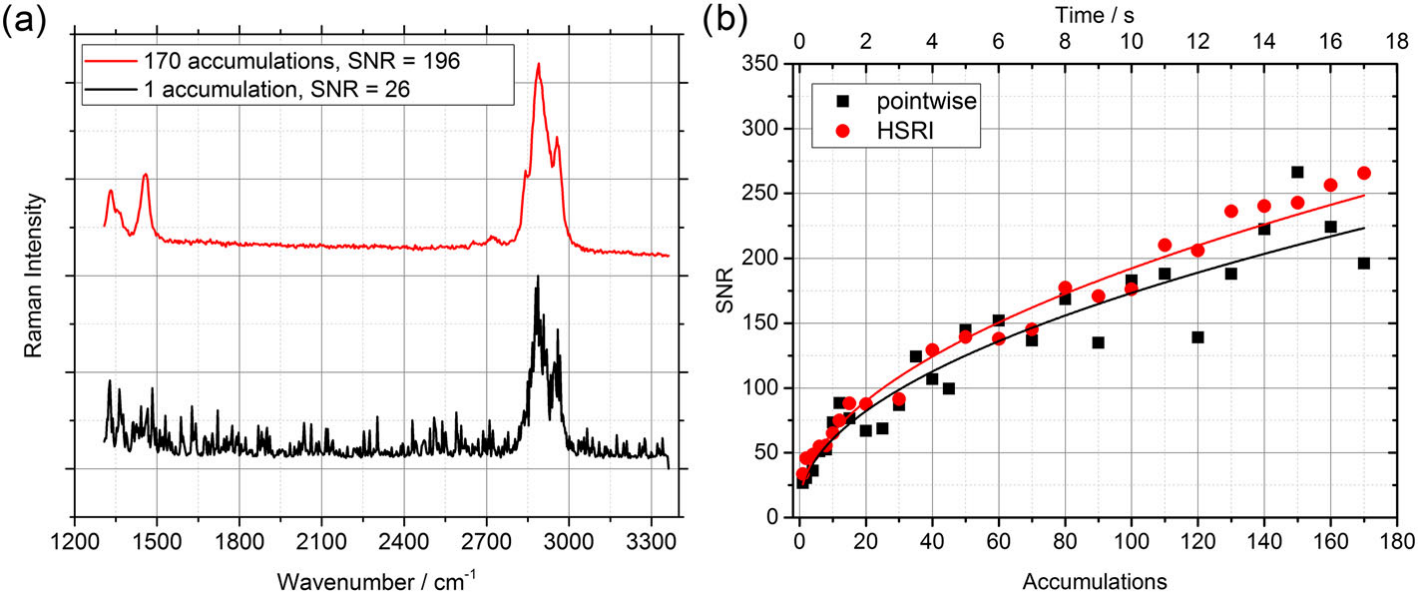
(a) Spectra of polypropylene acquired with the pointwise imaging system for two different number of accumulations with calculated signal‐to‐noise ratios (SNRs). (b) Signal to noise for both setups over the number of accumulations, that is, acquisition time. HSRI, hyperspectral Raman imaging [Colour figure can be viewed at wileyonlinelibrary.com]

To investigate the signal‐to‐noise performance of PI and HSRI, a PP plate was mounted at 15 m and measured with the systems. Both instrumentations work in a pulsed and time‐gated mode, meaning that after a laser pulse is emitted, a trigger signal is sent to the camera. After a set time delay of typical a few hundreds of nanoseconds (depending on the sample‐telescope distance), the gate of the intensifier is opened for 5 ns. This way, otherwise, interfering light sources can be suppressed, and the Raman signal from the sample can be maximized. The SNR in this study was calculated by using the mean intensity between 2,890 and 2,905 cm^−1^ (at the center of the C–H stretch vibrational band) divided by the standard deviation between 3,090 and 3,200 cm^−1^ (baseline noise). When only one‐shot measurements are performed, spectra tend to be of low quality as can be observed in Figure 4a. Therefore, usually more than one pulse is accumulated on the CCD in order to increase the SNR. With increasing number of accumulations, the quality of the spectra improves considerably.

Figure 4b shows the increase of SNR with increasing numbers of accumulation in the expected way of the square root of accumulations (fitted curves). For the HSRI, an area of pixels equaling the excitation area of the PI imager was averaged (28 mm^2^) in order to calculate the SNR. As visualized in Figure 4b, the PI imager and the HSRI show similar results in SNR. This favors the HSRI setup, when comparing the laser power impinging onto the sample. The laser sends out an energy of 50.8 mJ per pulse, which yields an averaged power of 0.5 W and considering a beam diameter of 6 mm results in power density of 1.77 W/cm^2^ for the PI system. The HSRI uses an expanded beam to an effective diameter of approximately 100 mm, which leads to a power density 6.4 mW/cm^2^, a 277‐fold decrease compared with the PI system. The possibility of using lower laser power densities is beneficial for practical use of such stand‐off Raman systems, where moderate power density levels are sought after due to eye‐safety concerns in civil and also military applications. Since the intensity of scattered Raman photons is directly proportional to the input of laser power onto the sample, the much lower power density for the HSRI should cause a significant drop‐off in SNR. Instead, a comparable SNR performance of both systems was observed. This can be explained by the higher optical throughput of the optical system, for example, higher throughput of the LCTF compared with the spectrograph, on one side and a difference in detection efficiency of the iCCD cameras. The PIMAX 1024RB used in the PI imager has a quantum efficiency of about 7.5%, whereas the GenIII intensifier of the PIMAX4 1024HBf used in the HSRI has one of 45%, six times more. Additionally, the étendue, a measure for the gathering power of an optical system, which is equal to the source emitting area multiplied by the solid angle from which the light is collected, is different. Due to the small diameter of the single fibers used in the bundle, the PI has an étendue of 0.27 mm^2^ sr, whereas the HSRI can use up to the full sensor size of the CCD camera, amounting to an étendue of 1.09 mm^2^ sr. This is an increase of a factor of 4 and shows one of the downsides of fiber coupled stand‐off Raman instruments.^28^ The throughput of the PI system could however be optimized by using a spectrograph with a F number more suitable to the used fiber bundle. Launching the f/2.2 fiber bundle into the f/10 spectrograph approximately 40% of the light are lost.

Additionally, it is especially useful if larger potentially contaminated or hazardous areas have to be scanned. The studied instrumentations behave differently for this task. The time consuming step for the PI is to move from point to point in order to map the area of interest. So the total measurement time scales with size of the investigated area and the spatial resolution defined by the excitation laser beam diameter. The time‐consuming step for HSRI is to collect spectral images at each desired Raman shift in order to build up the HSI cube. Here, the total measurement time scales with the number of spectral images necessary for spectroscopic determination of the analytes of interest that is the extension of the spectral range. Additionally, if the area under investigation is bigger than the FOV of the HSRI, further images have to be taken. Figure S1 shows a comparison of the total measurement time for both setups. It assumes a FOV for the HSRI of 70 × 70 mm^2^ at 15 m, the green and blue solid lines are the calculated time values for the respective prototype with the configuration used in this study. The dashed lines are calculated for a low spatial resolution for the PI and for a small number of spectral images for the HSRI. The dashed‐dotted lines represent the opposite situation, where high spatial resolution is needed for the PI system and a high number of spectral images are needed for the HSRI system. The semitransparent areas represent the possible or working span of both techniques (Figure S1).

Generally, the PI instrument will outperform the HSRI for small areas of interest, because only a few or, in the best case, a single measurement will suffice to complete the assessment of the target. For areas greater than 45 cm^2^, the HSRI starts to be faster than the pointwise acquisition; however, after reaching areas over 49 cm^2^, the HSRI has to retake another image outside its FOV, which creates a massive increase in time consumption reflected in the step of graph. Finally, for greater image sizes than approximately 200 cm^2^, the HSRI will always be faster than the PI. Overall, looking at the span stretched over the maxima of both techniques, the HSRI imaging speed scales more advantageously with time.

**Figure 5:**
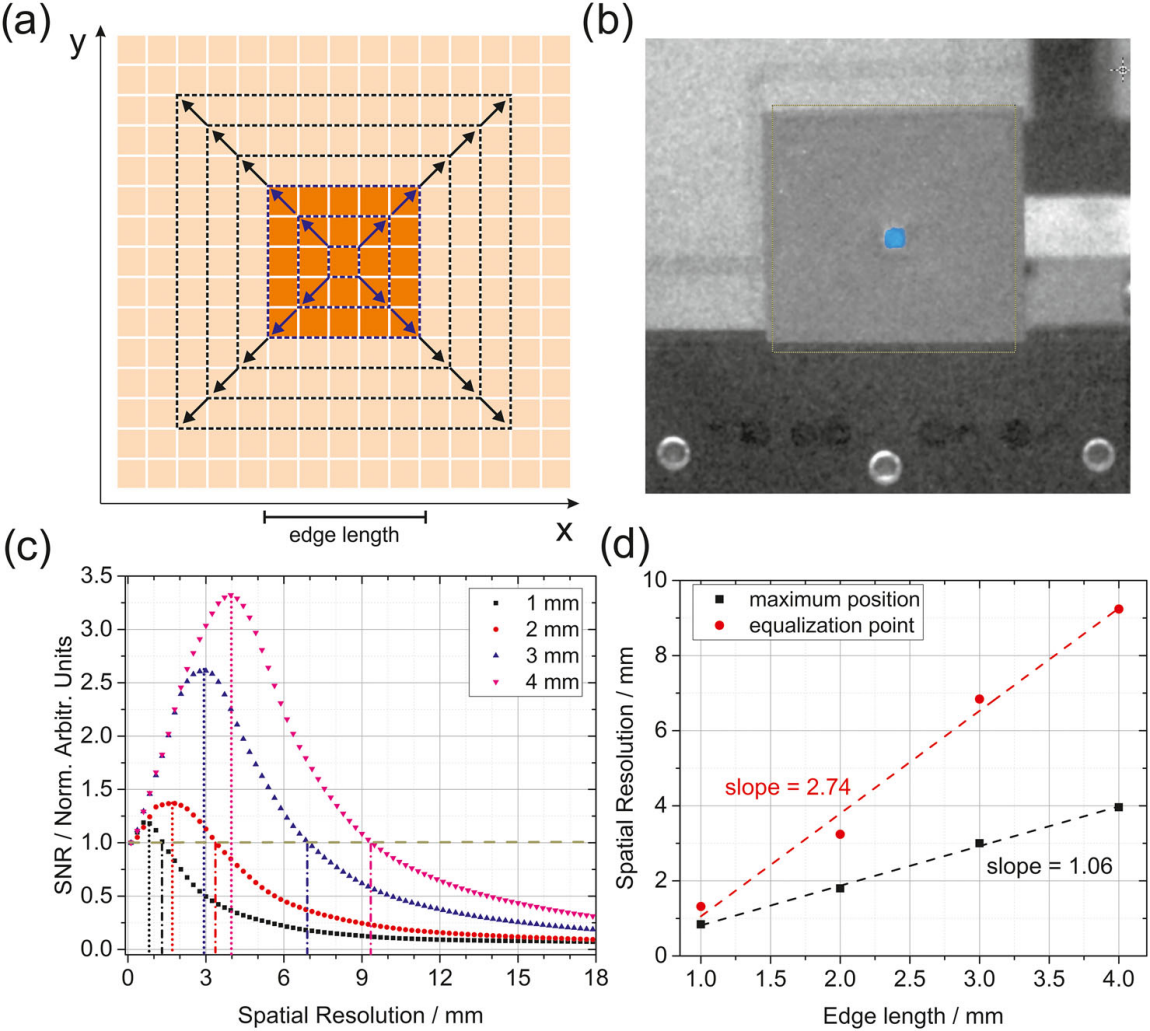
(a) Simplified drawing of the charge‐coupled device (CCD) chip with an arbitrary feature on top of it. The dashed squares indicate different spatial resolutions. (b) Grayscale image of the sample at 15 m with a blue overlay of the intensity of the sulfur band at 480 cm^−1^. (c) Signal‐to‐noise ratios (SNRs) calculated for the sulfur for different spatial averaging (simulating different spatial resolutions) and different feature sizes. The pointed lines are the position of the maximum SNR. (d) Spatial resolution for which the maximum of the SNR and the equalization point can be observed for different feature sizes, that is, edge lengths [Colour figure can be viewed at wileyonlinelibrary.com]

In order to highlight the importance of spatial resolution, samples with a sulfur feature of different sizes have been prepared by 3D printing polymer plates holding different sizes of cavities filled with sulfur powder. Square cavities with an edge length in the range of 1 to 4 mm and a thickness of 0.3 mm were measured at a distance of 15 m by employing 2‐s acquisition time per spectral snapshot (20 accumulations), 140‐ns gate delay and 5‐ns gate width using the HSRI system. The influence of spatial resolution was explored post measurement by choosing a central pixel inside the feature and averaging adjacent pixel spectra to decrease the spatial resolution. Then, the SNR was calculated through the maximum band intensity between 478 and 482 cm^−1^ (at the center of the sulfur band) divided by the standard deviation of the baseline between 510 and 580 cm^−1^ (baseline noise). The concept of this study is depicted in Figure 5a, where the reproduction of an arbitrary sample feature (dark orange) on an array detector (light orange) through the imaging optics is indicated. The stepwise variation of the spatial resolution is achieved by spatially averaging spectra of adjacent pixels. As long as the sample feature is bigger than the spatial resolution, spatial averaging (blue arrows in Figure 5a) will result in an increase of SNR following a square root function, because the noise in the baseline is reduced with every averaging step, but the signal level stays constant. In Figure 5c, the SNRs for different feature sizes are depicted in dependence of the spatial resolution, normalized to the first value for each series. In the beginning, the SNR increases according to the previous stated decrease of noise. When the spatial resolution surpasses the feature size, the situation changes dramatically (indicated by the black arrows in Figure 5a), as new pixels containing solely noise are added and the signal is diluted over the whole pixel area. This still leads to a reduction of noise; however, the signal intensity is also heavily reduced, which results in a significant reduction of SNR. The tipping point should coincide with the spatial resolution being of the same size as the sample feature, which was also observed in the experiments (Figure 5d, black curve). Another interesting point is the equalization point, the spatial resolution where the SNR of the different‐sized features drops down to the original value observed at the highest spatial resolution possible and after which a decrease in spatial resolution always results in lesser quality spectra. It is more than double the value of the feature size in the studied cases (Figure 5d, red curve). This shows in general the importance of having adequate spatial resolution, especially when small amounts of sample are to be detected on large background materials. The concept is also valid for other HSI techniques, but whiskbroom systems are more prone to difficulties, because the time‐consuming step is the measurement with adequate spatial resolution. The stand‐off HSRI shows its strength here, because producing adequate spatial resolution is not the time‐consuming task and can even be changed during the measurement and in postprocessing on a software basis.

### 3.4 Chemometric image analysis

**Figure 6:**
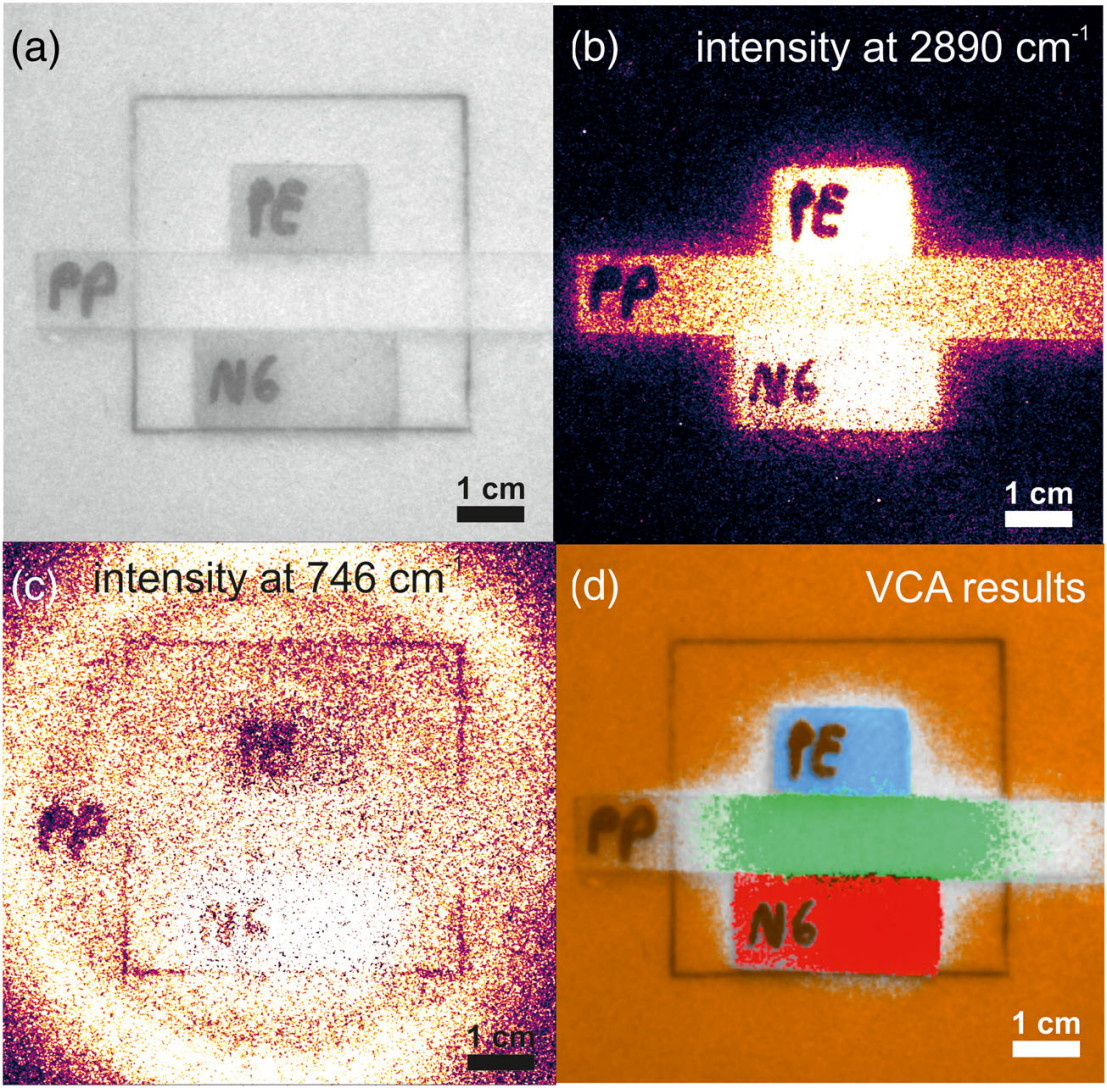
(a) Monochrome image of the sample consisting of the four polymers. (b) Intensity distribution at 2,850 cm^−1^ (polyethylene [PE]). (c) Intensity distribution at 746 cm^−1^ (polytetrafluoroethylene [PTFE]). (d) Overlay for vertex component analysis (VCA) endmembers correlation to the different polymers in different colors [Colour figure can be viewed at wileyonlinelibrary.com]

The HSRI approach usually leads to large datasets, which in most cases is not informative for the user nor required to solve the given analytical problem. Hence, efforts are made to simplify data interpretation by using a variety of algorithms to deconvolute and classify the recorded data in order to facilitate the extraction of the required information needed to provide a meaningful result. Linear unmixing algorithms are commonly used for hyperspectral datasets, when the presence of pure pixels can be assumed. Among these methods, VCA is advantageous because the VCA algorithm competes with state‐of‐the‐art methods while exhibiting a computational complexity between one and two orders of magnitude lower than the best available method.^25^ It additionally involves noise characterization in order to reduce the sensitivity to noise by applying a singular value decomposition. VCA calculates endmembers of the vertices of a simplex spanned by the spectral differences within the dataset. The endmembers represent the most varying spectra with nonnegative intensities and concentrations and can be assigned to chemical constituents in most cases. An example is given in Figure 6, where a sample consisting of four different polymers (PTFE, PE, PP, and nylon) is depicted (Figure 6a). It was placed at a distance of 15 m to the telescope and measured using the HSRI prototype. The intensity images of the bands 746 and 2,890 cm^−1^ are shown. The effects of polymer thickness and reflectivity can then be observed in Figure 6c. Since PTFE is the background material, the intensity at 746 cm^−1^ reflects the laser beam intensity distribution, except for the area blocked by the PE. In the case of PE, which is thicker and less transparent, a drop‐off in intensity can be observed. For the nylon, however, which is of the same thickness as the PE, a stronger signal is obtained, which is attributed to the fluorescence of the material, which results in an elevated baseline. The signal at 2,850 cm^−1^ is very intense for all polymers, except for PTFE, exhibiting the CH stretch vibration at this spectral region. Figure S2 provides a comparison of spectra of selected pixels of the respective polymer measured with the HSRI with a confocal Raman microscope. The VCA is able to find most pixels associated with the respective polymers, except towards some boundary regions between them, where because of mixed spectra the algorithm does not correlate correctly. Nevertheless, as shown in Figure 6d, VCA finds PE (marked in blue), PP (marked in green), nylon (marked in red), and PTFE (marked in orange). This fast classification of the image can prove useful to a variety of applications.

## 4 CONCLUSION

In this study, a comparison of a pointwise stand‐off Raman imager and a direct hyperspectral Raman imager in terms of achievable FOV, signal to noise, spectral and spatial resolution, and total measurement time is reported. The investigated prototypes differ in maximal possible FOV, which for the PI is limited by the numerical aperture of the optical fiber coupling the telescope with the spectrograph and for the HSRI by the FOV of the telescope and CCD size. The HSRI exhibits a FOV of roughly 70 × 70 mm, which is nearly 10 times more than the FOV of the PI. The stability of the spectral resolution of the tunable filter over the open aperture was investigated and determined to be better than 2 cm^−1^ for the central position and better than 4 cm^−1^ for the FWHM of the PTFE band at 746 cm^−1^. This proves the viability of the filter as a dispersive element for direct Raman imaging. The excitation beam has to be expanded in order to illuminate the whole scene for the HSRI, which leads to significantly lower power density at the sample. For the tested scenario, the difference in power density amounts close to 277‐fold increase with the PI imager. Nevertheless, the HSRI setup shows similar SNR values over the same measurement time, although the PI system would benefit substantially from a better matched fiber bundle‐spectrograph matching.

A significant contributor to the time needed for data acquisition is the size of the area to be investigated. A bigger FOV helps with screening larger areas faster. Inversely, the higher the spatial resolution of the imager has to be, the more time a mapping instrument would need, because the diameter of the excitation laser beam would need to be small and the number of mapping points would substantially increase. Spatial resolution is better suited for the HSRI, because the whole FOV of the collection optics can be used with a spatial resolution defined by the pixel size of the CCD chip. In time‐gated configuration like stand‐off applications, the required time is proportional to the repetition rate of the laser and inversely proportional to the number of accumulations, which holds true for both configurations.

In summary, two stand‐off Raman imagers were constructed and compared, one relying on mapping of the excitation laser beam, the other directly imaging the scene for single Raman shifts in order to create a HSI cube. The HSRI instrument showed better suitability for large area scanning, especially if a small number of spectral snapshots are required and offers a smaller electric and mechanic footprint, making it the better choice for mobile applications in the field.

## Supporting information


**Fig S1.** Total measurement time over image size at a target distance of 15 m. R_spat_ means spatial resolution of the PI, n_spec_ is the number of spectral images for the HSRI.
**Fig S2.** Comparison of stand‐off HSRI spectra and reference spectra collected with a Horiba LabRAM confocal microscope. All spectra were baseline corrected and normalized for better comparability. Nylon showed significant fluorescence during the measurement with the HSRI, so the characteristic of the Rayleigh filter is visible in the spectrum.
**Fig S3. a)** Example spectra of the PTFE plate at 15 m distance. **b)** Example of a Voigt profile fit for the band at 746 cm^‐1^. **c)** Spatial distribution of the central position of the PTFE band over the whole image. **d)** Spatial distribution of the FWHM of the PTFE band over the whole image. The black circle indicates the area of illumination by the laser beam.
