# Supplementary material for: Comparing mapping and direct hyperspectral imaging in stand‐off Raman spectroscopy for remote material identification
Source: J Raman Spectrosc. 2019 Apr 30;50(7):1034–43. doi: 10.1002/jrs.5607 (PMC6774338; doi:10.1002/jrs.5607)
Supplement: Supplementary file 1 — Fig S1. Total measurement time over image size at a target distance of 15 m. Rspat means spatial resolution of the PI, nspec is the number of spectral images for the HSRI. Fig S2. Comparison of stand‐off HSRI spectra and reference spectra collected with a Horiba LabRAM confocal microscope. All spectra were baseline corrected and normalized for better comparability. Nylon showed significant fluorescence during the measurement with the HSRI, so the characteristic of the Rayleigh filter is visible in the spectrum. Fig S3. a) Example spectra of the PTFE plate at 15 m distance. b) Example of a Voigt profile fit for the band at 746 cm‐1. c) Spatial distribution of the central position of the PTFE band over the whole image. d) Spatial distribution of the FWHM of the PTFE band over the whole image. The black circle indicates the area of illumination by the laser beam. [file JRS-50-1034-s001.docx]

**Supplementary Information**

**Comparing mapping and direct hyperspectral imaging in stand-off Raman spectroscopy for remote material identification**

*Christoph Gasser^1^, María González-Cabrera^2^, María José Ayora-Cañada^2^, Ana Domínguez-Vidal^2^, Bernhard Lendl^1,*^*

^1^ Institute of Chemical Technologies and Analytics, TU Wien, Vienna, Austria

^2^ Department of Physical and Analytical Chemistry, Universidad de Jaén, Campus Las Lagunillas, s/n 23071, Jaén, Spain

^*^Corresponding Author: bernhard.lendl@tuwien.ac.at


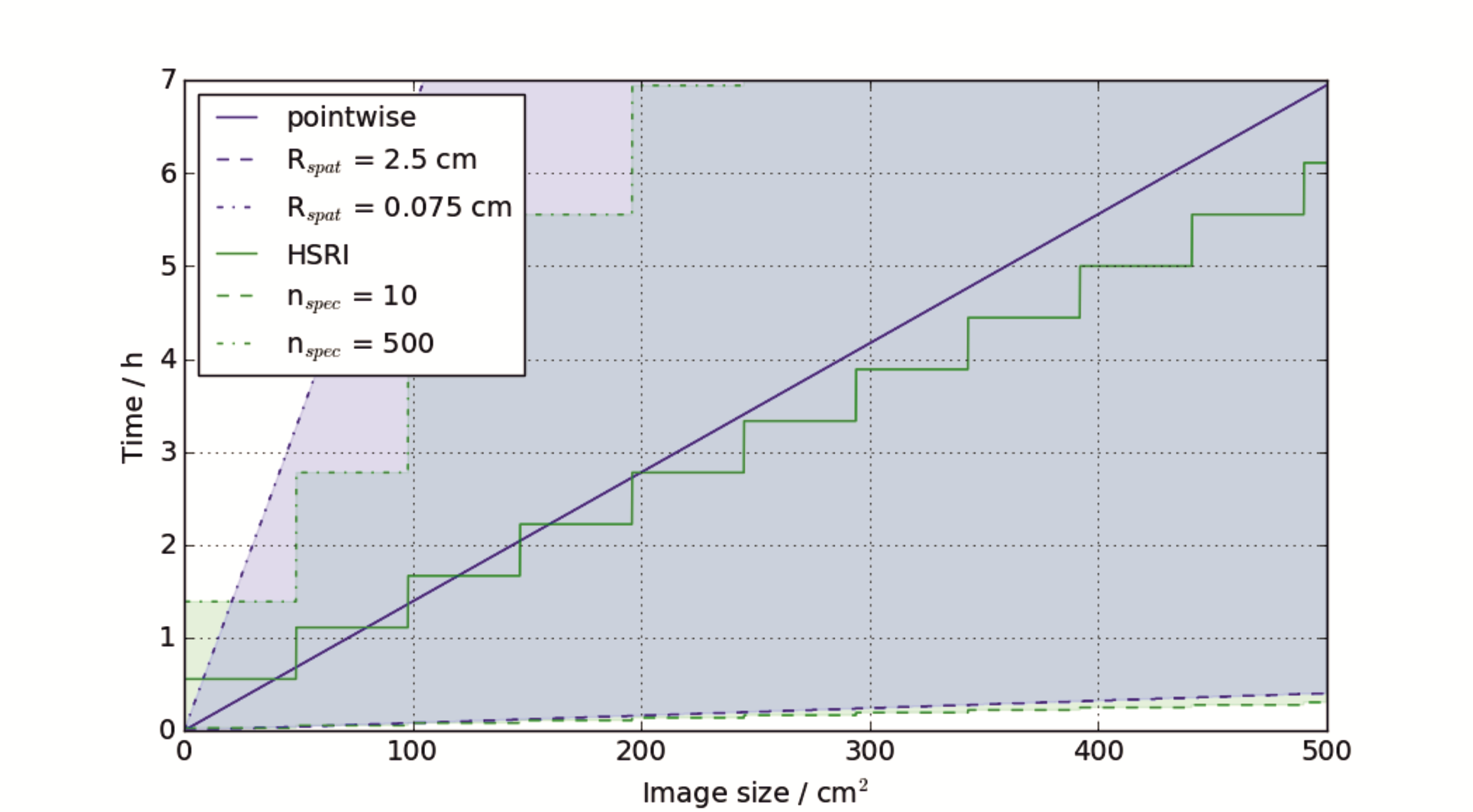


**Fig S1.** Total measurement time over image size at a target distance of 15 m. R_spat_ means spatial resolution of the PI, n_spec_ is the number of spectral images for the HSRI.

**
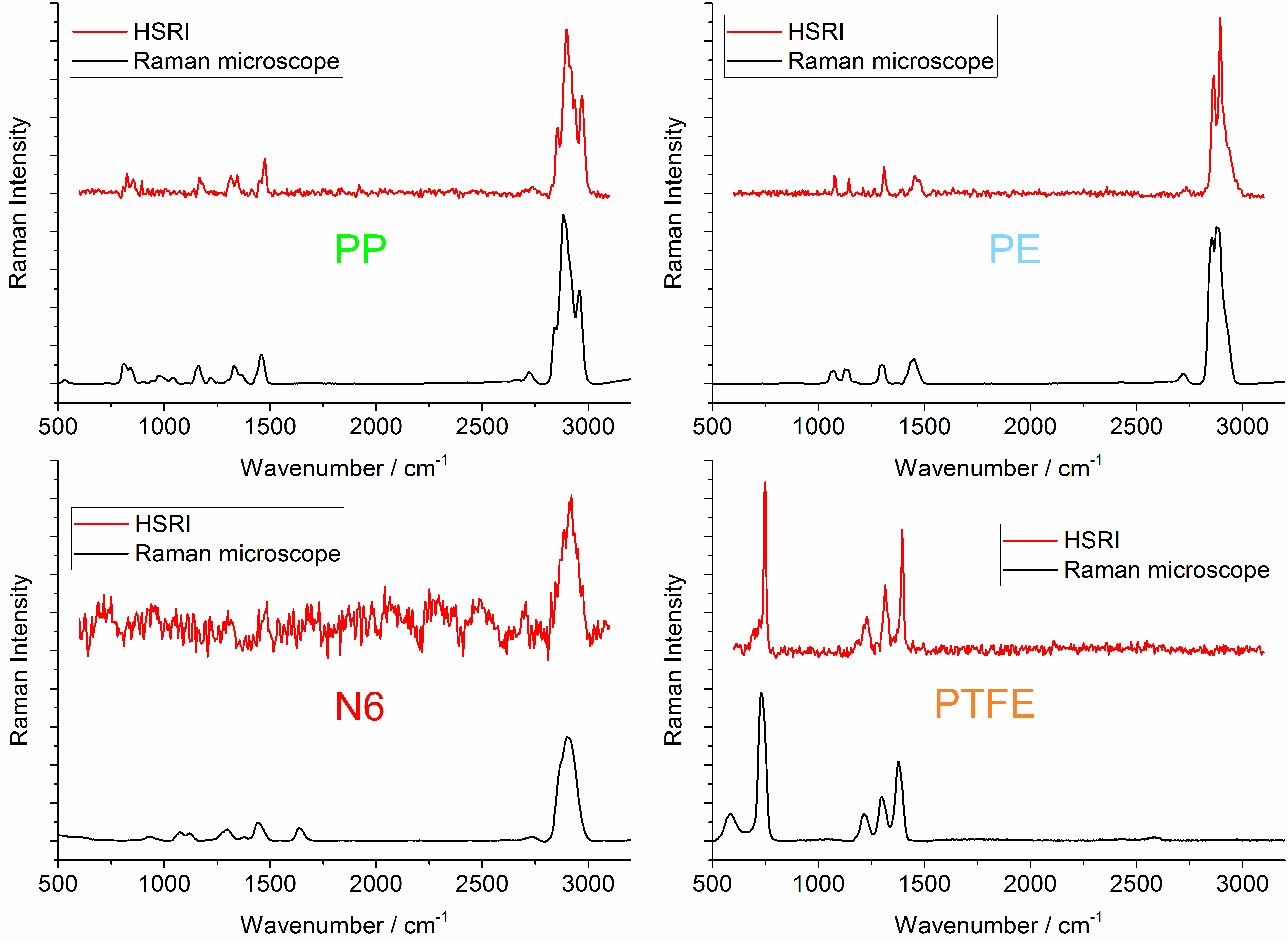
**

**Fig S2.** Comparison of stand-off HSRI spectra and reference spectra collected with a Horiba LabRAM confocal microscope. All spectra were baseline corrected and normalized for better comparability. Nylon showed significant fluorescence during the measurement with the HSRI, so the characteristic of the Rayleigh filter is visible in the spectrum.


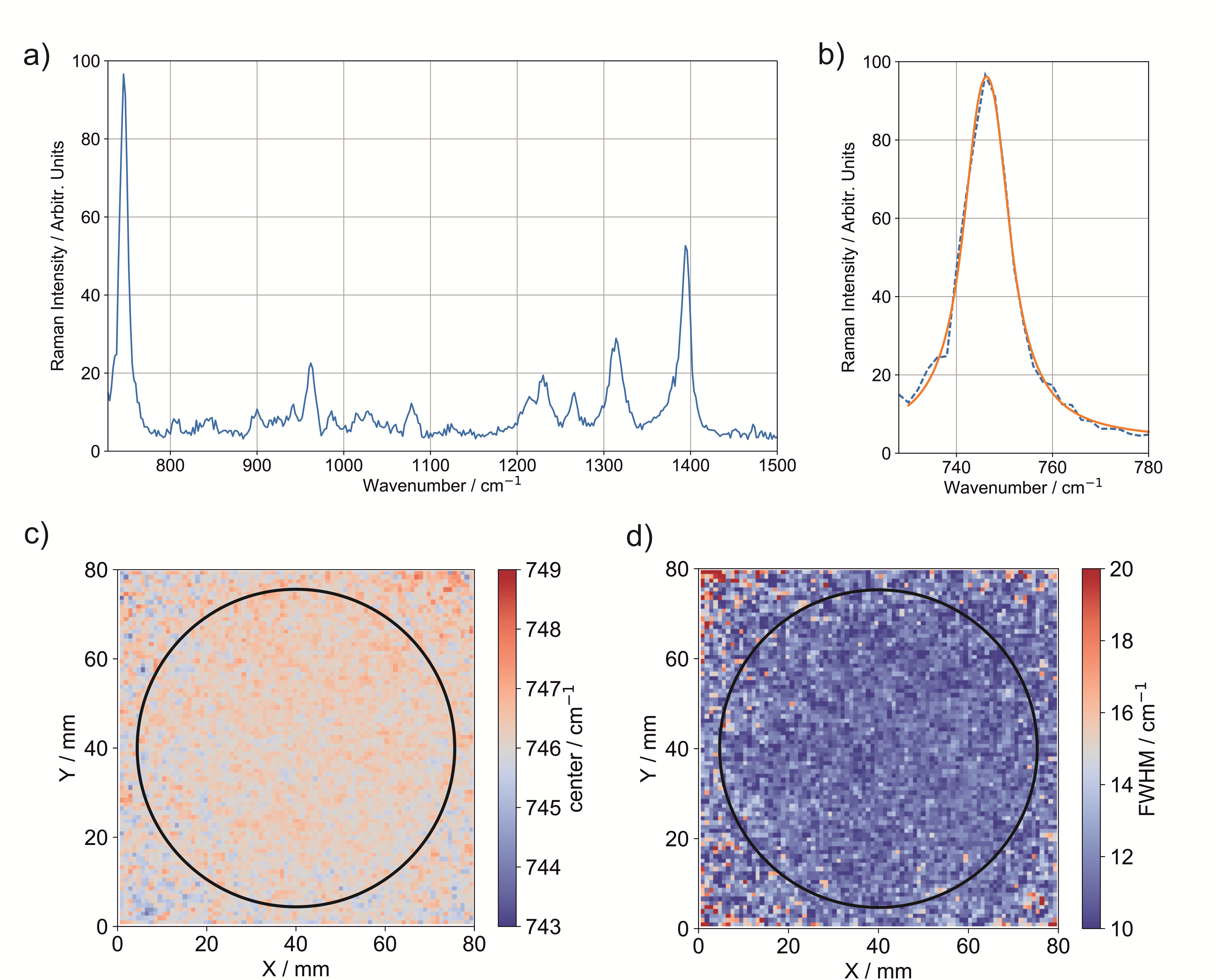


**Fig S3. a)** Example spectra of the PTFE plate at 15 m distance. **b)** Example of a Voigt profile fit for the band at 746 cm^-1^. **c)** Spatial distribution of the central position of the PTFE band over the whole image. **d)** Spatial distribution of the FWHM of the PTFE band over the whole image. The black circle indicates the area of illumination by the laser beam.
